# Ambler's Journal of Dental Operations

**Published:** 1849-04

**Authors:** 


*Ambler's Journal of Dental Operations.*—The importance to the dentist of having a book in which he can record the various peculiarities of the cases submitted to his treatment, and enter the operation, must be apparent to the least reflecting mind. The above work is well adapted to this purpose, and the date being left blank, it will answer as well for future as present use. We have before had occasion to commend this work to the profession, and we again take pleasure in calling their attention to it. There being an engraving of the teeth of both jaws on each page, the dentist can mark the operation he performs upon each tooth, which may be serviceable for future reference.

The above work may be obtained from Mr. J. G. Ambler, 26 Park Place, N. Y.

